# Copy number variation in the complement factor H-related genes and age-related macular degeneration

**Published:** 2011-08-06

**Authors:** Katharina E. Kubista, Nirubol Tosakulwong, Yanhong Wu, Euijung Ryu, Jaime L. Roeder, Laura A. Hecker, Keith H. Baratz, William L. Brown, Albert O. Edwards

**Affiliations:** 1 Department of Ophthalmology, Ludwig Boltzmann Institute for Retinology and Biomicroscopic Lasersurgery, Rudolf Foundation Clinic, Vienna, Austria; 2 Biomedical Statistics and Informatics, Mayo Clinic, Rochester, MN; 3 Laboratory Medicine and Pathology, Mayo Clinic, Rochester, MN; 4 Departments of Ophthalmology, Mayo Clinic, Rochester, MN; 5 Institute for Molecular Biology, University of Oregon, Eugene, OR

## Abstract

**Purpose:**

To determine the contribution of copy number variation (CNV) in the regulation of complement activation (RCA) locus to the development of age-related macular degeneration (AMD).

**Methods:**

A multiplex ligation-dependent probe amplification assay was developed to quantify the number of copies of *CFH, CFHR3, CFHR1, CFHR4, CFHR2,* and *CFHR5* in humans. Subjects with (451) and without (362) AMD were genotyped using the assay, and the impact on AMD risk was evaluated.

**Results:**

Eight unique combinations of copy number variation were observed in the 813 subjects. Combined deletion of *CFHR3* and *CFHR1* was protective (OR=0.47, 95% confidence interval 0.36–0.62) against AMD and was observed in 88 (82 [18.6%] with one deletion, 6 [1.4%] with two deletions) subjects with AMD and 127 (108 [30.7%] with one deletion, 19 [5.4%] with two deletions) subjects without AMD. Other deletions were much less common: *CFH* intron 1 (n=2), *CFH* exon 18 (n=2), combined *CFH* exon 18 and *CFHR3* (n=1), *CFHR3* (n=2), *CFHR1* (n=1), combined *CFHR1* and *CFHR4* (n=15), and *CFHR2* deletion (n=7, 0.9%). The combined *CFHR3* and *CFHR1* deletion was observed on a common protective haplotype, while the others appeared to have arisen on multiple different haplotypes.

**Conclusions:**

We found copy number variations of *CFHR3, CFHR1, CFHR4*, and *CFHR2*. Combined deletion of *CFHR3* and *CFHR1* was associated with a decreased risk of developing AMD. Other deletions were not sufficiently common to have a statistically detectable impact on the risk of AMD, and duplications were not observed.

## Introduction

Complement factor H (gene, *CFH;* protein, factor H) is the main inhibitor of the alternative pathway of the complement system [1]. Dysfunction of factor H is associated with increased liability to infections and chronic diseases, such as type II membranoproliferative glomerulonephritis, atypical hemolytic uremic syndrome, and age-related macular degeneration (AMD) [2].

**Figure 1:**
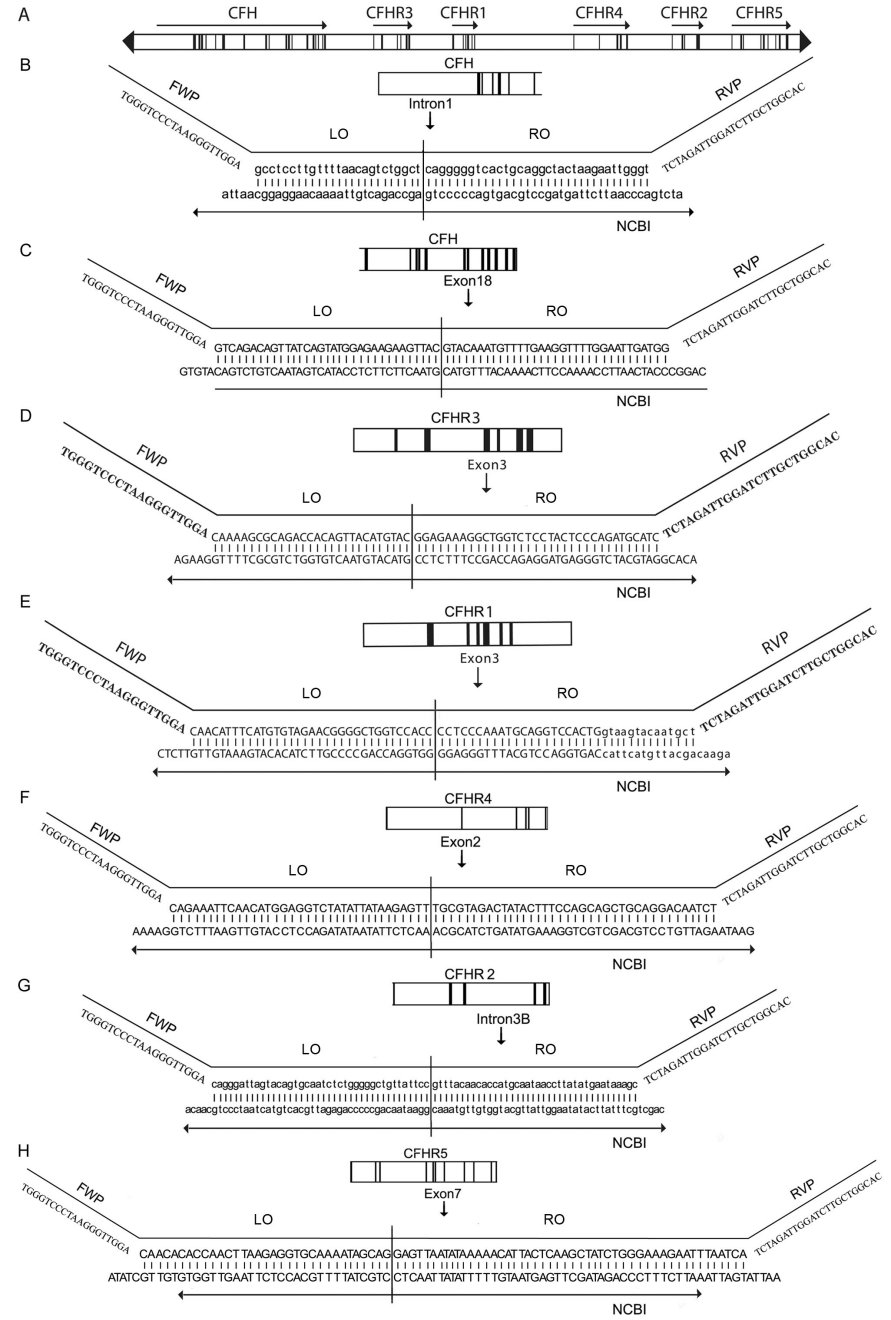
Multiplex ligation probe amplification strategy to quantify copy number variation across the regulation of complement activation locus, which is shown in panel **A**. It comprises complement factor H (*CFH*) and the five *CFH* related genes (*CFHR1–CFHR5*). Panels **B** to **H** describe the MLPA assays. The forward primer (FWP) is attached to the left oligonucleotide (LO), while the right oligonucleotide (RO) is attached to the reverse primer (RVP). The oligonucleotide pairs were designed to hybridize specifically to intron 1 of *CFH* (**B**), exon 18 of *CFH* (**C**), exon 3 of *CFHR3* (**D**), exon 3 of *CFHR1* (**E**), exon 2 of *CFHR4* (**F**), intron 3B of *CFHR2* (**G**), and exon 7 of *CFHR5* (**H**). Capital letters in the wild-type sequence from the National Center for Biotechnology Information (NCBI) correspond to exons, while lower case indicates introns. The MLPA procedure was described previously [12].

The 300,000 bp regulation of complement activation (RCA) locus on chromosome 1q32 contains *CFH* and five ancestrally related genes that lie in a head-to-tail arrangement (Figure 1). The five genes code for proteins that show sequence and structural homology to *CFH* and factor H. They are referred to as complement factor H-related (*CFHR*) genes and are numbered one through five. *CFH* and the five *CFHR* genes are thought to have developed by successive duplications within the RCA locus.

Genetic variation in genes encoding proteins for the alternative pathway of complement plays a major role in the development of AMD [3-8], which is the leading cause of vision loss in elderly individuals of the developed world [9]. Copy number variations in the form of deletions of *CFHR3* and *CFHR1* within the RCA locus have been reported to contribute to the development of AMD [10-13]. Because of the extensive linkage disequilibrium in the RCA locus, it has been difficult to determine if the CFHR proteins have a role in AMD independent of factor H, using statistical genetic approaches.

The amino acid sequence of the CFHR proteins is homologous to factor H, with the main difference being the presence or absence of different protein domains in the full length factor H. For example, CFHR3 is similar to factor H, except that it lacks the N-terminal protein domains that downregulate the alternative pathway. Thus, the protective effect of the common combined deletion of *CFHR3* and *CFHR1* is thought to occur through decreased competition of CFHR3 with factor H for binding to complement proteins [11,13]. CFHR1 was reported to inhibit the terminal complement pathway and thus might be involved in the pathogenesis of AMD [14]. CFHR4 interacts with native C-reactive protein (CRP), thereby enhancing opsonization via binding to CRP, which is elevated in the choroid and blood of subjects with AMD [15]. Also, variants in *CFHR2* and *CHR5* [16] have been demonstrated to be associated with AMD [17].

We report an analysis of copy number variation across the entire RCA locus and its possible contribution to the development of AMD. To overcome the extensive homology between the five *CFHR* genes, we developed an assay (Figure 1) using the multiplex ligation-dependent probe amplification (MLPA) technique [12,18-20]. We demonstrated that eight deletions of the *CFHR* genes segregate in the Caucasian population and we describe the haplotype backgrounds on which these deletions occur and their association with AMD.

## Methods

### Subjects

The study followed the tenets of the Declaration of Helsinki, was approved by the institutional review board of the Mayo Clinic (Rochester, MN), and written informed consent was obtained from all subjects after explanation of the nature and possible consequences of the study. The subjects were composed of the 813 self-reported Caucasian individuals described in Table 1. The ascertainment and characterization of the subjects has been reported [21]. Diagnosis was determined by review of fundus photographs as described previously [8,12,22-24]. Briefly, all subjects diagnosed with AMD had large drusen (≥125 µ) with sufficient drusen area to fill a 700-µ circle or more advanced findings. Controls had five or fewer hard drusen (<63 µ) without pigment changes or more advanced findings. Geographic atrophy and exudation were defined using the Wisconsin age-related maculopathy grading system [25]. Subjects have been graded multiple independent times by two individual highly qualified retina specialists [21]. Subjects with both neovascular and primary geographic atrophy, namely the development of geographic atrophy before the onset of exudation, were included in the analysis for each subtype. When a unique grade for each subject was required, the subjects graded “both” were added to the grade with a smaller number of subjects (geographic atrophy) to increase power [12,21,26].

**Table 1:** Demographic and clinical features of the subjects.

**Subjects**	**Number**	**Age (mean±standard deviation)**	**Male:Female ratio**
Controls	362	70.4±8.1	0.74
AMD subtotal	451	77.6±8.8	0.46
Early AMD	210	75.1±9.4	0.43
Geographic atrophy*	84	80.1±5.7	0.65
Exudative AMD	157	79.5±8.5	0.43
Total subjects	813	74.4±9.2	0.58

Twenty-two subjects who had both primary geographic atrophy and exudative age related macular degeneration (AMD; category “Both”) were included in the geographic atrophy group as described in methods.

### Multiplex ligation-dependent probe amplification

#### Design of oligonucleotides

**Figure 2:**
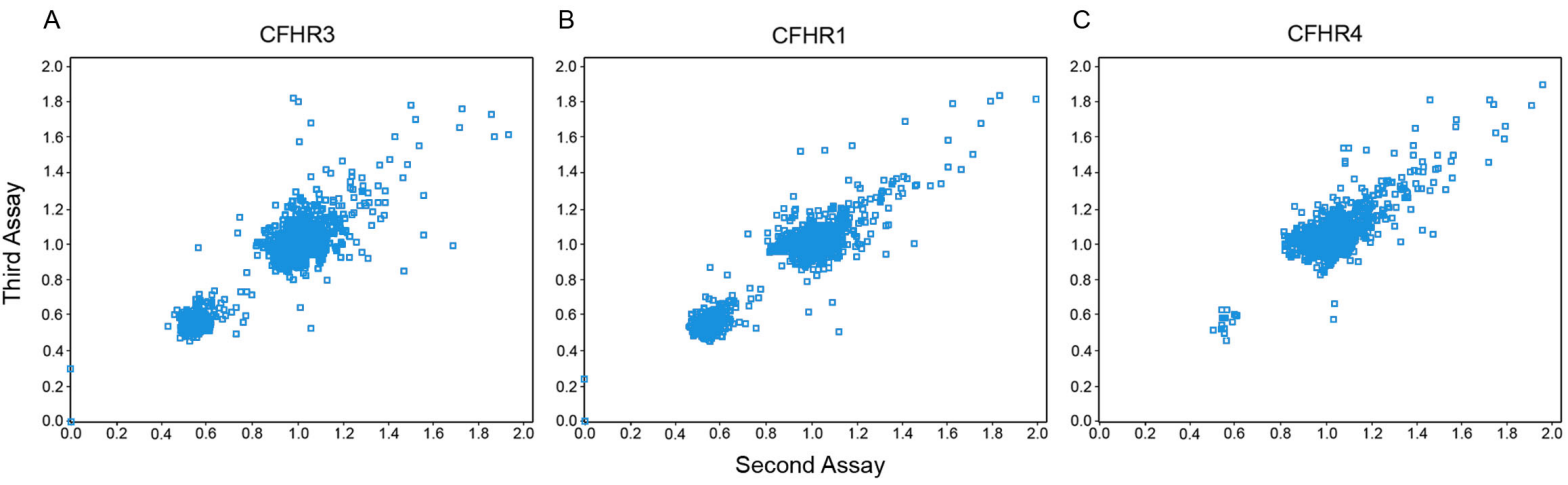
Scatter plots comparing the copy number estimates determined for the second and third MLPA assay on all subjects. Panel **A** shows the results for *CFHR3*, Panel **B** for *CFHR1*, and Panel **C** for *CFHR4*. The scatter plots show that the MLPA ratio criteria for homozygous deletion is 0.00 to 0.40, for heterozygous deletion is 0.41 to 0.80, for wild-type is 0.81 to 1.60, and for heterozygous duplication is >1.60. The largest cluster in each panel represents two copies, the second largest cluster one copy, and the subjects on the *y*-axis in panels **A** and **B** zero copies.

Oligonucleotides for MLPA were designed to bind specifically to *CFH* (intron 1), *CFH* (exon 18), *CFHR3* (exon 3), *CFHR1* (exon 3), *CFHR4* (exon 2), *CFHR2* (intron 3), and *CFHR5* (exon 7); Figure 1. Control oligonucleotides binding to the apoptosis-inducing factor mitochondrion-associated 1 (*PDCD8* (*AIFM1*)) on the X chromosome and synovial sarcoma translocation chromosome 18 (SS18) on chromosome 18 were also designed and included. The oligonucleotide pairs consisted of a left oligonucleotide (LO) and a right oligonucleotide (RO). The pairs were designed 1) to have their ligation point specifically at a known sequence variation of the homologous genes as described in Figure 1, 2) to have a 4-bp difference in length for each gene of interest, ranging from 100 to 132 bp, and 3) to have a GC content of 40%–60%. To allow quantitative analysis with capillary electrophoresis, the forward primer in the PCR was labeled with a fluorescent 6-carboxy-fluorescine (FAM) marker at the 5′ end. To avoid quenching of the fluorescent FAM marker by the G base, the standard MLPA forward primer [18,20] was redesigned and the sequence is shown in Figure 1. The oligonucleotides and the primers were obtained from Integrated DNA Technology (Coralville, IA; Table 2).

**Table 2:** Target sequences of the oligonucleotides used in the MLPA assay.

**Target Gene**	**Oligonucleotide side**	MLPA Oligonucleotides (5′ - 3′) hybridization sequences*	**Product length (bp)**
*CFH*	LO	gcctccttgttttaacagtctggct	100
(Intron 1)	RO	P-cagggggtcactgcaggctactaagaattgggt	
*CFHR3*	LO	CAAAAGCGCAGACCACAGTTACATGTAC	104
(Exon 3)	RO	P-GGAGAAAGGCTGGTCTCCTACTCCCAGATGCATC	
*CFH*	LO	GTCAGACAGTTATCAGTATGGAGAAGAAGTTAC	108
(Exon 18)	RO	P-GTACAAATGTTTTGAAGGTTTTGGAATTGATGG	
*CFHR1*	LO	CAACATTTCATGTGTAGAACGGGGCTGGTCCACC	112
(Exon 3)	RO	P-CCTCCCAAATGCAGGTCCACTGgtaagtacaatgct	
*CFHR4*	LO	CAGAAATTCAACATGGAGGTCTATATTATAAGAGTT	116
(Exon 2)	RO	P-TGCGTAGACTATACTTTCCAGCAGCTGCAGGACAATCT	
*CFHR2*	LO	cagggattagtacagtgcaatctctgggggctgttattcc	120
(Intron 3B)	RO	P-gtttacaacaccatgcaataaccttatatgaataaagc	
*CFHR5*	LO	CAACACACCAACTTAAGAGGTGCAAAATAGCAG	124
(Exon 7)	RO	P-GAGTTAATATAAAAACATTACTCAAGCTATCTGGGAAAGAATTTAATCA	
*PDCD8*	LO	CGCAAATACAACAGGTATCAGAACTGCTGGCCCCAGATTAAG	128
(Exon 8)	RO	P-CTTCAGATGGTGAACTCTGTGCACTTCCACCCATGCAGTCACCT	
*SS18*	LO	GGCAATCATATGATGGGTCAGAGACAGATTCCTCCCTATAGAC	132
(Exon 6)	RO	P-CTCCTCAACAGGgtaagattccatttggaaaatttgctacttaatgt	

*Sequences within an exon are shown in upper case, while sequences in introns are presented in lower case. The LO begins with the forward primer sequence 5′-TGG GTC CCT AAG GGT TGG A-3′ followed by the sequence shown in the table. The 5′-end of the RO is phosphorylated (P) and the sequence shown in the table is followed by the complement of the reverse primer 5′-TCT AGA TTG GAT CTT GCT GGC AC-3′. Figure 1 and the methods provide additional details.

#### Assay conditions for MLPA

We performed MLPA according to the MLPA protocol using the MLPA EK kit (MRC-Holland, Amsterdam, Netherlands) and 50 ng DNA per sample. Four control samples (two male and two female) without deletions of *CFH* and *CFHR1–5*, based on the MLPA assay, and a water (no DNA) control were included in each experiment. Hybridization, ligation, and the setup for amplification of the MLPA assay were performed as described previously [12]. Amplification conditions were 30 cycles at 95 °C for 30 s, 60 °C for 30 s, and 72 °C for 60 s, followed by 20 min at 72 °C and a hold at 4 °C.

#### Assay conditions for capillary electrophoresis

Two microliters of the amplified products were diluted (1:30) in 58 µl of distilled water, of which 2 µl was plated into a well with 18 µl of a mixture of 1 part formamide Hi-Di (Applied Biosystems, Foster City, CA) and 0.015 parts GeneScan 120 LIZ size standard (Applied Biosystems). Capillary electrophoresis was performed on an Applied Biosystems 3730 DNA Analyzer (Applied Biosystems) as described previously [12]. If the detection threshold was crossed, the amplified product was diluted in distilled water (1:50) and 2 µl of the dilution was used for capillary electrophoresis. If the fluorescent intensities were too low, 2 µl of the pure amplified product was used for capillary electrophoresis.

#### Data analysis

GeneMapper Software v4.0 (Applied Biosystems) was used to perform fragment sizing with the internal 120 LIZ size standard, automated peak calling, and peak normalization [27]. The nine peak heights of the nine probes (*CFH1* [*CFH* intron 1], *CFH18* [*CFH* exon 18], *CFHR3*, *CFHR1*, *CFHR4*, *CFHR2*, *CFHR5*, *PDCD8,* and *SS18*) of each sample were exported for further data analysis. The peak heights were normalized by dividing each peak height by the sum of all nine peak heights in each sample. Each normalized peak height was divided by the means of that peak height in the four control samples to standardize the peak heights to the control samples included in every run. The ratio for the probes *CFH1*, *CFH18*, *CFHR3*, *CFHR1*, *CFHR4*, *CFHR2*, *CFHR5,* and *PDCD8* of every sample was generated by dividing the normalized and standardized peak heights of the probe by the normalized and standardized peak height of the control probe *SS18* in that sample. The probe of the X chromosome (*PDCD8*) was used as a control for unintended interchange of samples and as a model of deletions. Each assay run was calculated separately due to possible interassay variations, as recommended [18]. The person performing the assay and the calculations was masked to the ophthalmologic diagnosis of the subjects [12].

#### Replication of MLPA assay results

The MLPA assay was performed three times on all samples. A sample was defined as having “failed” if i) a result was not repeated a second time, ii) the *SS18* control probe did not result in two alleles, and iii) the gender of the sample did not match the number of alleles of the control probe *PDCD8* (i.e., a male subject should only have one copy, while a female subject should have at least two copies of the *PDCD8* gene). Selected samples were repeated using the TaqMan quantitative PCR assay (Applied Biosystems).

### TaqMan assay

A TaqMan assay was performed to independently confirm the copy number variation of eight randomly selected samples. Specific genomic targets in *CFH* (intron 1), *CFH* (intron 18), *CFHR3* (exon 3), *CFHR1* (exon 3), *CFHR4* (intron 2), *CFHR2* (intron 3), and *CFHR5* (exon 7) were amplified using the TaqMan Gene Expression Master Mix (Applied Biosystems) combined with the specific target gene assay mix consisting of a 0.25-mM TaqMan MGB probe and 0.9 mM of each PCR primer [12]. Forward primer 5′-TGT TTT GCC AAC GGA CCT ATT TAG T-3′ and reverse primer 5′-GCC CAT TAA TAG GAG CAT TTA TTT TGC T-3′ was employed for *CFHR3*. Forward primer 5′-ACA TCT CCA ATT TAG ATC CTT TGA TTA ACC A-3′ and reverse primer 5′-GCA TTT TCT TAG TGA ATA AGC AAA GAT TTA AAA ACA-3′ was used for *CFHR1*. The TaqMan assay used for *CFH* (intron 1) was localized at 194890541; for *CFH* (exon 18) it was localized at intron 18 at 194976628; for *CFHR4* it was at intron 2 at 195139068; for *CFHR2* it was at intron 3 at 195188714; and for *CFHR5* it was at exon 7 at 195234027. TaqMan probes and primers were obtained from Applied Biosystems. All reactions were performed in triplicate, as described previously [12]. The values obtained for *CFH1*, *CFH18*, *CFHR3*, *CFHR1*, *CFHR4*, *CFHR2*, and *CFHR5* copy numbers were normalized to the endogenous control gene RNase P and quantified relative to the copy number of control samples using the ΔΔCT method [28].

### Genotyping

Single nucleotide polymorphisms (SNPs) tagging common haplotypes across the RCA locus were genotyped as described previously [8,12,24,26,29].

### Statistical analyses

All SNPs and copy number variant assays were noted to be in Hardy–Weinberg equilibrium (p>0.05). Single variant analyses on genotype distributions were performed in SAS version 9.13 (SAS institute, Cary, NC) using logistic regression assuming a log-additive genetic model where variants were coded as 0, 1, or 2 for the number of minor alleles or deletions. Fisher’s exact tests were performed also on genotype distributions. Haplotype analyses on SNPs across the RCA locus and the occurrence of *CFHR3*, *CFHR1*, *CFHR4,* and *CFHR2* copy number variation was performed using haplo.stats packages (Mayo Foundation for Medical Education and Research) in R. To investigate the effect of polymorphisms on AMD subtypes, each subtype was compared to the control. Age is confounded with diagnosis (i.e., the cases are older than the controls and age is a risk for AMD), thus correction for age might have unpredictable effects; all analyses were performed with and without correction for age and gender [26]. Nominal p-values are reported.

## Results

### Development of a new MLPA assay

Ten samples from our previous paper [12] and an additional two male and two female control samples were chosen for evaluating the new MLPA assay (Figure 1). The new MLPA assay gave the same results for the four loci in our previous assay (*CFHR3*, *CFHR1*, *SS18,* and *PDCD8)* [12], which was reproducible upon running the assay three times. The reproducibility of the capillary electrophoresis was demonstrated by repeating this step twice. MLPA was also performed on the four control samples with different oligonucleotide combinations, and no unwanted probe amplification (e.g., from the CFHR loci omitted from a given assay) was observed. The products amplified by each of the nine amplicons in the MLPA assay were electrophoresed on agarose gels, and single bands of the expected product sizes were observed.

### Validation of ratio change thresholds for copy number variation in the MLPA assay

**Figure 3:**
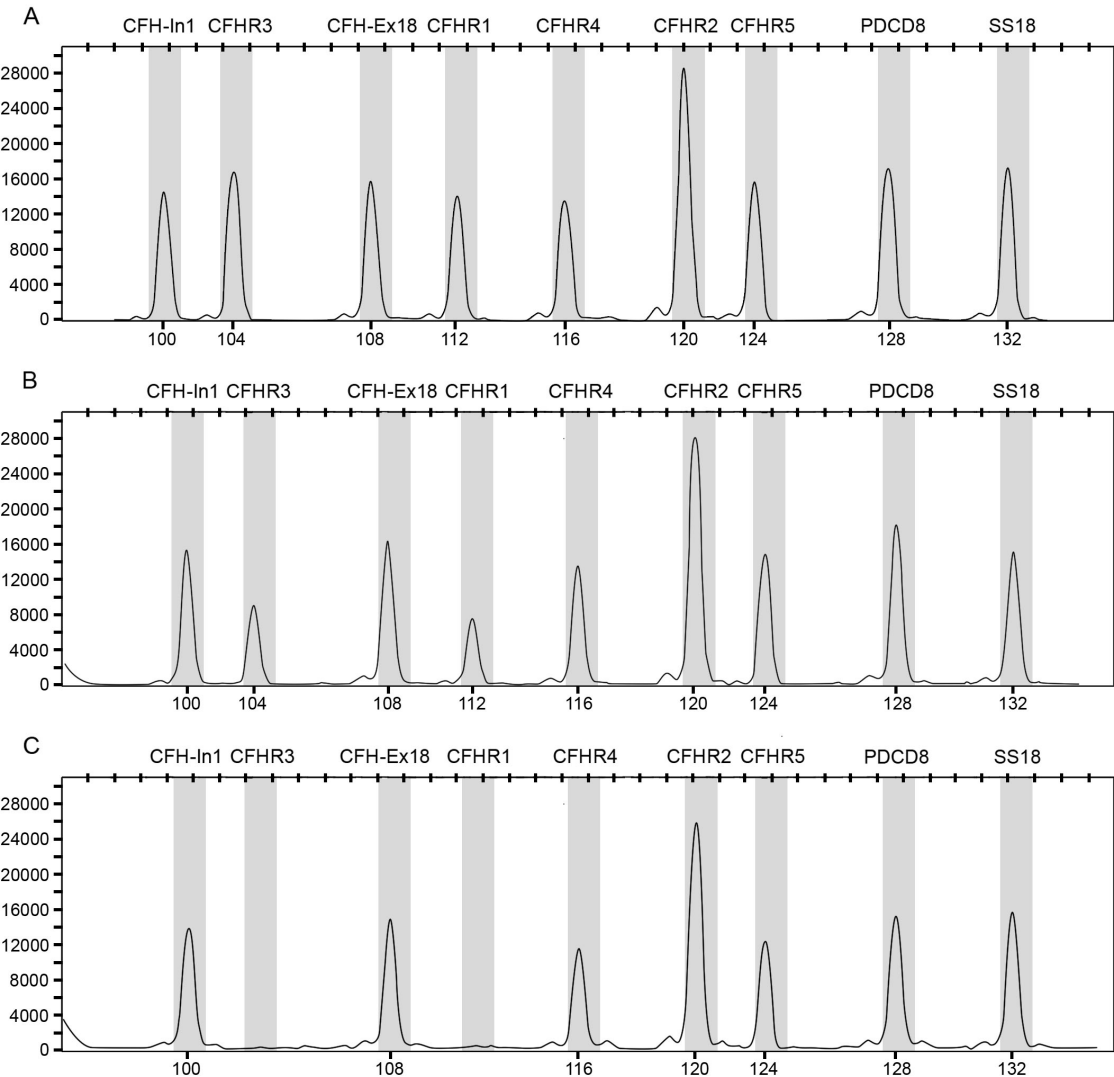
Example of an MLPA copy number assay. Raw fluorescent intensity (*y*-axis, relative fluorescence units) observed during capillary electrophoresis are illustrated. The first peak represents the amplicon of *CFH* intron 1 with a product size of 100 bp (x-axis, bp), followed by the other probes as illustrated. The ratio of peak height of all probes to *SS18* determines the copy number (see methods). *CFHR2* always has a high peak, which is taken into account with normalization. Therefore, this does not show a duplication of *CFHR2*. Panel **A** shows a wild-type subject, panel **B** a subject heterozygous for deletion of both *CFHR3* and *CFHR1*, and panel **C** a subject homozygous for deletion of both *CFHR3* and *CFHR1*. The X-chromosome marker (*PDCD8*) shows that all three subjects are females.

Scatter plot diagrams comparing the second and third independent assays of *CFHR3, CFHR1*, and *CFHR4* of all samples were used to define our MLPA ratio criteria (Figure 2). Based on this evidence and the replication using TaqMan assays, the standard MLPA ratio criteria for homozygous deletion (≤0.40), heterozygous deletion (≤0.80), and heterozygous duplication (>1.60) were employed throughout the study. Examples of raw data are shown in Figure 3.

### Copy number variation observed and association with AMD

Eight unique combinations of deletions were observed. The most common was the previously reported combined deletion of *CFHR3* and *CFHR1* (Table 3). The second most common was the combined deletion of *CFHR1* and *CFHR4* (Table 3). More rare deletions are presented in Table 4. Unexpectedly, no duplications were observed. We observed a significant protective effect of the combined deletions of *CFHR3* and *CFHR1* on risk of having AMD in both adjusted for age and gender and unadjusted calculations (OR=0.47, 95% CI 0.36–0.62). The effect was similar among different AMD subtypes compared to the controls (OR=0.51, 95% CI 0.36–0.73 for early AMD, and OR=0.43, 95% CI 0.31–0.62 for advanced AMD). The 15 observed combined deletions of *CFHR1* and *CFHR4* (eight cases and seven controls) did not show a significant impact on risk of having AMD (Table 3 and Table 4). The other deletions were also observed in a similar proportion of cases and controls, except for *CFHR2* which was seen in six cases and one control (Table 4).

**Table 3:** Common copy number variation observed and the association with AMD.

**Deletion**	**Minor allele frequency in all 813 subjects (%)**	**Final deletion count**	**Number subjects (%)**	**Control n (%)**	**AMD cases n (%)**	**Early AMD n (%)**	**Geographic atrophy n (%)**	**Exudative AMD n (%)**	**Advanced AMD‡ n (%)**	**OR (95% CI) §**	**Log-additive p-value**	**Log-additive p-value age, gender adjusted**
***CFHR3* and *CFHR1***	15	2	25 (3.1)	19 (5.4)	6 (1.4)	5 (2.4)	0 (0)	1 (0.7)	1 (0.4)	0.47 (0.36–0.62)	1.35E-07	1.24E-07
		1	190 (23.9)	108 (30.7)	82 (18.6)	37 (17.9)	11 (13.1)	34 (22.5)	45 (19.1)			
		0	579 (72.9)	225 (63.9)	354 (80.1)	165 (79.7)	73 (86.9)	116 (76.8)	189 (80.4)			
		Total	794* (100)	352 (100)	442 (100)	207 (100)	84 (100)	151 (100)	235 (100)			
***CFHR1* and *CFHR4***	1.3	2	0 (0)	0 (0)	0 (0)	0 (0)	0 (0)	0 (0)	0 (0)	0.73 (0.26–2.04)	0.5498	0.8081
		1	15 (2.5)	7 (3)	8 (2.2)	3 (1.8)	0 (0)	5 (4.1)	5 (2.6)			
		0	582 (97.5)	227 (97)	355 (97.8)	165 (98.2)	73 (100)	117 (95.9)	190 (97.4)			
		Total	597† (100)	234 (100)	363 (100)	168 (100)	73 (100)	122 (100)	195 (100)			

* The total for combined deletions of *CFHR3* and *CFHR1* is 794, because 19 subjects had other copy number variations and are not included in this analysis. †The total for combined deletions of *CFHR1* and *CFHR4* is 597, because 216 subjects had other copy number variations. ‡Advanced AMD is all subjects with geographic atrophy and/or exudative AMD. § This is the odds ratio (OR) for being a case rather than a control by adding one more deletion, assuming additivity on the log scale. Thus, the OR for two deletions compared to no deletion is the square of the OR for one deletion.

**Table 4:** Subjects with *CFH* intron 1 and exon 18, *CFH* exon 18 with *CFHR3*, *CFHR3* only, *CFHR1* only, combined *CFHR1* and *CFHR4*, and *CFHR2* deletions.

**Final deletion count**						**Demographics**		**Diagnosis**	
***CFH*** intron 1	***CFH*** exon 18	***CFHR3***	***CFHR1***	***CFHR4***	***CFHR2***	**Gender**	**Age**	**Affectation**	**Subtype**
1	0	0	0	0	0	F	77	Control	
1	0	0	0	0	0	F	66	AMD	Early
0	1	0	0	0	0	M	82	AMD	Exudative
0	1	0	0	0	0	M	72	Control	
0	1	1	0	0	0	F	69	Control	
0	0	1	0	0	0	M	65	Control	
0	0	1	0	0	0	M	85	AMD	Exudative
0	0	0	1	0	0	F	70	Control	
0	0	0	1	1	0	F	66	AMD	Exudative
0	0	0	1	1	0	F	43	AMD	Early
0	0	0	1	1	0	F	79	AMD	Early
0	0	0	1	1	0	F	89	AMD	Exudative
0	0	0	1	1	0	M	68	AMD	Exudative
0	0	0	1	1	0	M	88	AMD	Exudative
0	0	0	1	1	0	F	63	Control	
0	0	0	1	1	0	F	71	AMD	Early
0	0	0	1	1	0	M	66	Control	
0	0	0	1	1	0	F	86	AMD	Exudative
0	0	0	1	1	0	F	82	Control	
0	0	0	1	1	0	F	61	Control	
0	0	0	1	1	0	F	65	Control	
0	0	0	1	1	0	F	71	Control	
0	0	0	1	1	0	M	79	Control	
0	0	0	0	0	1	M	81	AMD	Early
0	0	0	0	0	1	F	90	AMD	Exudative
0	0	0	0	0	1	M	78	AMD	Exudative
0	0	0	0	0	1	F	84	AMD	Early
0	0	0	0	0	1	F	65	AMD	Early
0	0	0	0	0	1	M	68	Control	
0	0	0	0	0	1	M	71	AMD	Exudative

### Haplotype studies

Six common haplotypes were observed in the RCA locus, following previous reports [10-12,30]. Three haplotypes carried the Y402H polymorphism and increased the risk of AMD (R1, R2, and R3), one haplotype was neutral for AMD risk (N), and two haplotypes were protective (P1 and P2). The deletion of *CFHR3* and *CFHR1* was always present on a haplotype most similar to P2 (Appendix 1 and Appendix 2). SNP rs6677604 tagged the combined deletion of *CFHR3* and *CFHR1*, as reported previously [12]. The rs6677604 GG genotype occurred in 571 of 579 (99%) subjects without the combined deletion, the GA genotype in 189 of 189 (100%) subjects heterozygous for the combined deletion, and the AA genotype in 25 of 25 (100%) subjects homozygous for the combined deletion. The combined *CFHR1* and *CFHR4* deletions and the *CFRH2* only deletions occurred on multiple independent haplotypes and were not tagged by a single SNP.

## Discussion

We developed and validated a new MLPA assay to enable determination of the frequency and patterns of copy number variation in *CFH* and the five *CFHR* genes across the RCA locus. The MLPA assay was performed on 813 subjects with and without AMD. The well known combined deletion of *CFHR3* and *CFHR1* was observed on 15% of chromosomes in this study and was highly protective for AMD as reported by us and others previously (Table 3) [10-13,30]. We were able to verify that rs6677604 tags the combined *CFHR3* and *CFHR1* deletion in a larger group of subjects than reported previously [10,12,30]. So far no other tagging SNP has been found for these copy number variations.

Copy number variation in the human genome is associated with many diseases [31-36]. It has been suggested that copy number variations account for more nucleotide variations than do SNPs [37]. Due to their size copy number variations often encompass functional DNA sequences and can sometimes disrupt them [38] or lead to a protective effect. The combined deletion of *CFHR3* and *CFHR1* that is protective in AMD [10-13,30] is associated with an increased risk for atypical hemolytic uremic syndrome [14,39,40]. The combined deletion of *CFHR1* and *CFHR4* has only been described as an increased risk for the atypical hemolytic uremic syndrome [40]. Apart from the combined deletion of *CFHR3* and *CFHR1* and of *CFHR1* and *CFHR4*, we observed an additional six patterns of deletions. However, these were not sufficiently common to have a detectable effect on AMD risk. Deletion of each *CFHR* gene was observed, except for *CFHR5*. No duplications were detected, as would be expected if the recombination events were common recurrent events.

Zhang et al. [17] also reported that there was a significant association between variants of *CFHR2* and *CFHR5* and AMD risk and showed that a haplotype spanning *CFH* (including the Y402H *CFH* variant), *CFHR4*, and *CFHR2* was associated with the greatest risk of neovascular AMD (p<10^−6^). Narendra et al. [16] identified five different heterozygous sequence changes in *CFHR5* and suggested that the mutant T allele in exon 4, which was significantly higher in controls than in AMD patients (p<0.0001) and leads to a codon change at Asp169Asp, might be associated with a reduced risk of developing AMD. In a study of renal disease (with persistent microscopic hematuria, recurrent macroscopic hematuria, glomerulonephritis, and progressive renal failure), Gale et al. [41] found a rare internal duplication of exon 2 and exon 3 in *CFHR5* that may account for the substantial proportion of renal disease in Cyprus and called it “CFHR5 nephropathy” [41]. We designed our oligonucleotide pair for MLPA within exon 7 of *CFHR5* and did not observe copy number variation within this region of *CFHR5* in our subjects. Narendra et al. [16] would not have detected duplication of exons 2 and 3, and the effect of this duplication on AMD is as yet unknown. Thus, variation in the copy number of the *CFHR* genes is associated with atypical hemolytic uremic syndrome and the CFHR5 nephropathy, but a definitive association with AMD is still lacking.

We have observed extensive association between SNPs across the RCA locus, including each of the *CFHR* genes, since our original report in 2005 [8]. However, we have been unable to demonstrate an effect independent of *CFH* using statistical genetic approaches. The mechanism through which the genetic variation across the RCA contributes to AMD remains a subject of active investigation. Because of the tendency of polymorphisms across this 300,000-bp and six-gene region to be co-inherited (linkage disequilibrium), independent effects of the genetic variation in this region is difficult to assess using statistical genetic approaches. However, haplotypes can be estimated across the region, and the functional evaluation of these ancestral blocks of DNA has proven insightful [24].

A small number of common haplotypes are found across the *CFH* gene (Appendix 1) [6-8,12,42,43]. Depending on the number of polymorphisms included in the estimation of haplotypes, there is a group of risk haplotypes (R1, R2, and R3) that contain the Y402H polymorphism and increase the risk of AMD. Two of these haplotypes (R1, R2) appear identical across the *CFH* gene, while R3 has some differences after exon 14. The protective haplotypes (P1 and P2) appear to protect against AMD, while the neutral (N) haplotype does not have an impact on AMD risk.

We previously reported that each of the common haplotypes (R1, P1, P2, and N) had a distinct influence on the activation and levels of complement in human blood [24]. Notably, the P1 haplotype which carries the I62V polymorphism reduced complement activation in human blood [24] and biochemical functional studies [44,45], while the R1, P2, and N haplotypes had no effect on blood levels of complement activation [24]. These two observations are consistent with a local role for the combined deletion of *CFHR3* and *CFHR1* in decreased complement activation in Bruch’s membrane and the choroid. Experimental studies are needed to confirm the hypothesis noted earlier that deletion of *CFHR3* reduces competition with factor H inhibition of the alternative pathway of complement. Our results suggest that any such effect may occur on the surface of molecules of the RPE, Bruch’s membrane, and choroid.

The combined deletion of *CFHR3* and *CFHR1* was a consistent feature of the P2 haplotype. The combined deletion of *CFHR3* and *CFHR1* appears to have arisen on the P2 haplotype, which can be defined by a C at rs3766404 and an A at rs6677604 (Appendix 1 and Appendix 2). All but 22% of the combined deletion of *CFHR3* and *CFHR1* reside on this haplotype. The combined deletion of *CFHR3* and *CFHR1* was always observed on a core region of the P2 haplotype TAGAAGG from rs1061170 through rs1065489 (Appendix 2). The less common haplotypes on which the combined deletion of *CFHR3* and *CFHR1* reside could have arisen through recombination on the 5′ region of *CFH* (e.g., rs800292 or the functional I62V polymorphism) and SNPs far downstream of *CFH*. Notably, the results provide further evidence that the protective effect mediated by the combined deletion of *CFHR3* and *CFHR1* is independent of the enhanced cofactor activity of the P1 haplotype provided by the I62V polymorphism [24,44,45]. We found that the full P2 haplotype was found in combined deletion homozygotes of *CFHR3* and *CFHR1* with a frequency of 53%, similar to reports by Spencer et al. [30] (47%) and Hageman et al. [11] (63%).

Raychaudhuri et al. [42] showed that Y402H is in linkage disequilibrium with rs10737680 and the combined *CFHR3* and *CFHR1* deletion. By univariate analysis, they showed that each marker has a significant association with AMD. When they conditioned on Y402H alone, they demonstrated that the combined deletion effect was still present. However, when they conditioned on rs10737680, the statistical strength of the protective effect of the combined deletion was alleviated. Also, Neale et al. performed logistic regression to test if copy number variations are associated with disease and found that the *CFHR1* deletion has a strong association with AMD but that it does not describe an independent association [46]. However, Hughes et al. confirmed by logistic regression analysis that the protective haplotype, which includes the combined *CFHR3* and *CFHR1* deletion, confers a significant independent effect on AMD [10]. Li et al. [47] demonstrated that dissection of complex disease susceptibility loci is very challenging and that even if the Y402H variant is strongly associated with AMD, it is unlikely to be the only major determinant of disease susceptibility in this region. The strong linkage disequilibrium in this region limits statistical methods to distinguish between alternative sets of associated SNPs. The important question that we are trying to address is does the P2 haplotype confer a protective effect beyond not having the Y402H polymorphism. The P2 haplotype has no residual impact on disease risk after statistical analysis conditioning on the risk haplotype [42,46]; however, we know from functional studies (e.g., the clear impact of I62V on complement levels) [24] that statistical analysis does not always capture the functional variants. Thus, a definitive answer cannot be excluded without functional studies.

In summary, we observed that the combined deletion of *CFHR3* and *CFHR1* is the most common copy number variation across the RCA locus. The deletion is found on the protective P2 haplotype, and we have confirmed that it can be efficiently tagged by the single nucleotide polymorphism rs6677604. The seven other deletions we observed are rare and appear to have arisen on different haplotypes, suggesting that direct genotyping of the deletions is needed for their detection.

## Appendix 1. Common haplotypes across the RCA locus and haplotypes on which deletions were observed.*

*The combined CFHR1 and CFHR4 deletion was observed in 15 subjects on the following haplotypes in descending frequency: a recombinant haplotype between R3 and either R1, R2 or P2, a recombinant R1/P1 haplotype, P1, and a recombinant N/P1. The seven subjects with R2 deletions occurred on 4 different haplotypes: P1, R1, a recombinant N/R3, and R3. Note that 1 means present and 0 deleted. † The combined CFHR3 and CFHR1 deletion is a consistent feature of the P2 haplotype. ‡ The haplotype frequencies represent the absolute frequency of the haplotype in the samples. To access the data, click or select the words “Appendix 1.” This will initiate the download of a compressed (pdf) archive that contains the file.

## Appendix 2. Common haplotypes across the RCA locus and haplotypes in combined deletion homozygotes of CFHR3 and CFHR1.

* This is the P2 haplotype as described in Appendix 1. To access the data, click or select the words “Appendix 2.” This will initiate the download of a compressed (pdf) archive that contains the file.
